# A 15-Gene Signature and Prognostic Nomogram for Predicting Overall Survival in Non-Distant Metastatic Oral Tongue Squamous Cell Carcinoma

**DOI:** 10.3389/fonc.2021.587548

**Published:** 2021-03-09

**Authors:** Muyuan Liu, Litian Tong, Bin Liang, Xuhong Song, Lingzhu Xie, Hanwei Peng, Dongyang Huang

**Affiliations:** ^1^ Department of Head and Neck, Cancer Hospital of Shantou University Medical College, Shantou, China; ^2^ Department of Anesthesiology, Cancer Hospital of Shantou University Medical College, Shantou, China; ^3^ Department of Cell Biology and Genetics, Key Laboratory of Molecular Biology in High Cancer Incidence Coastal Chaoshan Area of Guangdong Higher Education Institutes, Shantou University Medical College, Shantou, China; ^4^ Research Center of Translational Medicine, Second Affiliated Hospital of Shantou University Medical College, Shantou, China

**Keywords:** gene signature, oral tongue squamous cell carcinoma (OTSCC), prognosis, overall survival (OS), nomogram

## Abstract

**Background:**

Oral tongue squamous cell carcinoma (OTSCC) is a devastating tumor with poor prognosis. There is an urgent need for reliable biomarkers to help predict prognosis and guide treatment for OTSCC. In the current study, we aimed to develop a robust multi-gene signature and prognostic nomogram to predict the prognosis of patients with non-distant metastatic OTSCC.

**Methods:**

OTSCC-related differentially-expressed genes were screened from The Cancer Genome Atlas (TCGA) database. Univariate Cox regression based on 1,000 bootstrap replicates, LASSO regression and stepwise multivariate Cox regression were utilized to develop a novel multi-mRNA signature for predicting overall survival in OTSCC. The concordance index, area under receiver operating characteristic (ROC AUC) and calibration curve were employed to assess the prediction capacity of the novel multi-gene model. In addition, a prognostic nomogram was constructed to facilitate the clinical use of the fitted model. The Kaplan-Meier with log-rank test was employed to assess differences in overall survival.

**Results:**

We successfully established a novel 15-mRNA prognostic model for predicting overall survival of non-distant metastatic OTSCC, involving *ADTRP, ITGA3, RFC4, CCDC96, CYP2J2, NELL2, SPHK1, SPAG16, HBEGF, S100A9, EGFL6, ADGRG6, PDE4D, ABCA4*, and *CTTN*. The prediction ability of this 15-gene signature was independent of other clinicopathological factors, with an HR of 11.5 (95% CI: 4.70–28.3). Moreover, internal validation by bootstrap analysis yielded a C-index of 0.849, with a 3-year AUC of 0.907 and 5-year AUC of 0.944, which implied excellent prediction accuracy of the fitted model. In addition, external validation by using the GEO dataset (GSE41116) yielded a C-index of 0.804, with a 3-year AUC of 0.868 and 5-year AUC of 0.855, which also indicated good prediction ability of the 15-gene model. Finally, a prognostic nomogram integrating risk group, grade, T stage and N stage was established.

**Conclusion:**

Our results demonstrate our 15-gene signature was independently associated with overall survival in non-distant metastatic OTSCC. Moreover, the prognostic nomogram integrating the 15-gene signature and clinicopathological factors has potential to be developed as a prognostic tool.

## Introduction

Oral tongue squamous cell carcinoma (OTSCC) is the most common cancer in the oral cavity, comprising 90% of all cases of oral malignancies (1, 2). Despite advances in treatment for OTSCC, the 5-year overall survival rate remains poor (3). Although the TNM staging system is of great value for risk stratification of OTSCC, it has imprecise prognostic predicting capability to guide patient management due to the heterogeneity of the disease characteristics. Thus, new prognostic models are essentially needed to identify the high-risk patients with OTSCC and consequently improve personalized treatment. Moreover, a precise and quantitative prognostic model is essential and valuable for patient counseling.

An increasing amount of evidence has demonstrated that aberrant expression of certain messenger RNAs (mRNA) is closely related to the prognosis of cancer patients and could be used as molecular biomarkers for the prognostic evaluation and identification of potential high-risk patients (4). In recent years, integration of an increasing number of molecular biomarkers into a single model is being established for prognostic evaluation (5–8). However, a multi-mRNA prognostic signature and nomogram for OTSCC patients are still lacking.

The Cancer Genome Atlas (TCGA) is one of the prominent databases containing genomic, transcriptomic, epigenetic, proteomic, and clinical information for different types of cancer. In the current study, we identified significant cancer-related genes that are differentially-expressed between non-distant metastatic OTSCC samples and normal tongue tissue samples in TCGA. Then, we utilized univariate Cox regression based on 1,000 bootstrap replicates, the least absolute shrinkage and selection operator (LASSO) regression model and the multivariate Cox regression model to establish a reliable and precise multi-mRNA-based prognostic signature for predicting overall survival of non-metastatic OTSCC patients. Simultaneously, a prognostic nomogram was established to facilitate the clinical use of the multi-mRNA signature. In addition, the C-index, calibration plot and time-dependent ROC curve were used to evaluate the ability of the model to predict prognosis.

## Materials and Methods

### Data Source and Identification of Differentially Expressed Oncogenes

First, for the training set, clinical and gene expression data of 13 normal tongue tissues and 127 OTSCC tissues was downloaded from TCGA database (https://www.cancer.gov/about-nci/organization/ccg/research/structural-genomics/tcga). For the external validating set, gene expression data of 28 OTSCC tissues was downloaded from the GEO database (GSE41116) (https://www.ncbi.nlm.nih.gov/gds/?term=GSE41116). Clinical data of these 28 OTSCC patients was downloaded from cBioPortal for Cancer Genomics (https://www.cbioportal.org). Next, differential expression analysis was employed to identify differentially-expressed genes based on mRNA expression differences having an absolute |logFC| >1 and *p* < 0.05. In addition, any differentially-expressed genes with an average expression value less than 100 were removed. Then, oncogenic signature enrichment analysis was performed using Metascape (http://metascape.org/gp/index.html#/main/step1) to identify the differentially-expressed cancer-related genes. These differentially-expressed cancer-related genes served as candidate genes for further regression analysis and prognostic model building.

### Establishment and Validation of the 15-Gene Signature Prognostic Model and Nomogram

In the present study, the correlation between overall survival (OS) and differentially-expressed cancer-related gene expression levels were studied using univariate Cox regression based on 1,000 bootstrap replicates. Those differentially-expressed cancer-related genes found to be statistically significant factors by univariate analysis were included in the LASSO-penalized Cox regression analysis to screen for independent prognostic genes and avoid over fitting. Then, stepwise multivariate Cox hazard ratio regression was performed to select the best model for predicting overall survival of non-distant metastatic OTSCC. Subsequently, an mRNA-based prognostic risk score was calculated by combining the expression values of prognostic genes and their regression coefficients from the multivariate Cox regression model. The risk-score calculation formula was as follows:

$$\text{Risk score}=\Sigma_{i=1}^{n}Expi*\beta i$$

Here, n represents the number of prognostic genes, Expi represents the expression value of prognostic gene i, and βi represents the regression coefficient of gene i from the multivariate Cox regression analysis. By using the median risk score as a cutoff value, OTSCC patients were divided into high-risk and low-risk groups, and the association between the prognostic gene signature and overall survival was investigated based on the risk groups.

A time-dependent receiver operating characteristic (ROC) curve was constructed and the area under the curve (AUC) was calculated to assess the predictive power of the prognostic model. Internal validation and external validation for the prognostic gene signature were performed using the C-index, and a calibration curve was drawn based on the bootstrap method. Co-expression analysis was employed to assess the collinear relationship of the prognostic genes. Finally, a nomogram for predicting overall survival of a M0 OTSCC patient was constructed using the risk group based on the prognostic gene model and clinical prognostic factors such as histologic grade and clinical stage. The calibration curve was studied to assess the performance of the prognostic nomogram.

### Statistical Analysis

All statistical analyses were performed with R software and packages from the Bioconductor project. Univariate Cox regression based on 1,000 bootstrap replicates was performed to identify single significant factors of survival outcomes. Differentially-expressed genes found to be statistically significant by univariate analysis were included in the LASSO-penalized Cox regression analysis to screen for independent prognostic genes. Then, stepwise multivariate Cox hazard ratio regression was performed to establish a prognostic gene signature for predicting overall survival of OTSCC. The area under the ROC curve (AUC) was used as an accuracy index to identify the best combination of multiple bio-markers. The predictive ability of the 15-gene signature and prognostic nomogram was estimated by using the calibration curve. Overall survival times were estimated by the Kaplan-Meier method. Kaplan-Meier survival curves were generated, and the statistical significance was evaluated by the log rank test. All significance tests were two-sided, and a *P* < 0.05 was considered significant.

## Results

### Clinical Characteristics of Oral Tongue Squamous Cell Carcinoma Patient Cohorts in The Cancer Genome Atlas and GEO

For the training cohort, clinical and RNA sequencing data of 13 normal tongue tissues and 127 OTSCC tissues was downloaded from TCGA (https://www.cancer.gov/about-nci/organization/ccg/research/structural-genomics/tcga). In this cohort, we included 83 male and 44 female patients with a median age of 57 years (range from 19 to 87 years). Fifteen patients were AJCC stage I, 23 patients were stage II, 28 patients were stage III, and 61 patients were stage IV. Moreover, 70 patients had pathologically-positive cervical lymph nodes. All those cases had no distant metastasis. For the external validating cohort, a gene expression matrix of 28 OTSCC tissues was downloaded from GEO (GSE41116) (https://www.ncbi.nlm.nih.gov/gds/?term=GSE41116) (9). Clinical data of these 28 OTSCC patients was downloaded from cBioPortal for Cancer Genomics (https://www.cbioportal.org). The gene expression matrix and clinicopathological characteristics of the validating set is shown in **Supplementary Data 1**. In this cohort, there were 18 male and 10 female patients with a median age of 60 years (range from 26 to 85 years). Six patients were AJCC stage II, 13 patients were stage III, and 9 patients were stage IV. Consistent with the training cohort, all the cases in the validating cohort had no distant metastasis. Clinicopathological characteristics of the training cohort and testing cohort are showed in **Table 1**.

**Table 1:** Clinicopathological characteristics of the patients included in the training set and validating set.

Clinicopathological parameters	Training Set (TCGA dataset, n = 127)	Validating Set (GEO dataset, n = 28)
Sex		
Male	83 (65.4%)	18 (64.3%)
Female	44 (34.6%)	10 (35.7%)
Age	57 (y, median)	60 (y, median)
T Stage		
T1-2	67 (52.7%)	6 (21.4%)
T3-4	60 (47.3%)	22 (78.6%)
N Stage		
N^−^	57 (44.9%)	9 (32.1%)
N^+^	70 (55.1%)	19 (67.9%)
Grade		
Well	17 (13.4%)	2 (7.1%)
Moderate	86 (67.7%)	19 (67.9%)
Poor	24 (18.9%)	7 (25.0%)

### Identification of a 15-Gene Signature for Predicting Overall Survival for Non-Distant Metastatic Oral Tongue Squamous Cell Carcinoma

#### Candidate Screening for Differentially Expressed Cancer-Related Gene

In total, 6,111 differentially-expressed genes were identified using edgeR. Then genes with an average expression less than 100 were excluded, resulting in 2,579 differentially-expressed genes being retained. Next, oncogenic signature enrichment analysis by Metascape was performed and 901 differentially-expressed cancer-related genes were identified for further regression analysis.

### Univariate Cox Regression Based on 1,000 Bootstrap Replicates

We employed univariate Cox regression based on a bootstrap resampling technique (1,000 replicates) to generate a hazard ratio (HR) with both 95% bias-corrected and accelerated (BCa) bootstrap confidence intervals, as well as the 95% percentage (Perc) bootstrap confidence interval. Both BCa and Perc 95% confidence intervals that did not include zero were considered statistically significant and were incorporated in the LASSO regression analysis. The bootstrap univariate regression results are listed in **Supplementary Table 1**. In the end, 41 out of 901 differentially-expressed cancer-related genes were identified as prognostic factors for OTSCC (**Table 2**).

**Table 2:** Forty-one significantly differentially-expressed cancer-related genes selected by univariate Cox regression analysis.

Gene	HR	Perc		BCa	
		95% CI_lower	95% CI_upper	95% CI_lower	95% CI_upper
**KLF7**	2.051967	1.181178	4.013881	1.169583	4.006894
**TPTEP1**	0.489758	0.254359	0.833391	0.282388	0.892341
**ADTRP**	2.185642	1.280026	4.108594	1.101236	3.597496
**MAGIX**	0.395979	0.196065	0.691413	0.211579	0.735964
**ITGA3**	2.003327	1.103321	3.727782	1.025672	3.542512
**RFC4**	1.887744	1.034734	3.317223	1.078374	3.395053
**CCDC96**	0.430815	0.241363	0.775666	0.24291	0.776434
**SLC16A1**	2.302277	1.254623	4.446845	1.143051	3.997884
**BUB1**	1.883744	1.068125	3.44771	1.082299	3.56022
**ECT2**	2.064229	1.193709	3.845979	1.167009	3.664635
**MYO10**	1.872354	1.074121	3.459864	1.077746	3.487003
**STARD4**	1.97469	1.129966	3.52473	1.120917	3.502698
**ADAM8**	1.91557	1.077973	3.428193	1.054494	3.28236
**CYP2J2**	0.492253	0.27153	0.903332	0.262146	0.885367
**ANKRD6**	0.527813	0.283685	0.905571	0.28838	0.91253
**ZNF43**	0.418219	0.215498	0.759636	0.227062	0.774721
**NELL2**	1.942154	1.131742	3.661527	1.131149	3.648482
**HMMR**	2.0045	1.141503	3.624981	1.086084	3.533556
**CENPE**	1.8295	1.033575	3.431939	1.050664	3.445997
**SPHK1**	0.49007	0.25464	0.873303	0.253429	0.867494
**GMNN**	1.931726	1.095112	3.652783	1.102352	3.672541
**DPT**	0.519485	0.281145	0.896171	0.291404	0.945245
**FOXF2**	0.403354	0.207124	0.688939	0.204161	0.680017
**SPAG16**	0.484103	0.250436	0.877999	0.247645	0.86013
**PRC1**	1.782683	1.061899	3.250286	1.029389	3.162917
**BTBD19**	0.537572	0.287423	0.970207	0.292695	0.981974
**HBEGF**	1.844453	1.055062	3.368863	1.035218	3.279491
**LIMD2**	0.431411	0.227756	0.752626	0.255372	0.821784
**MAD2L1**	1.831203	1.083429	3.399503	1.010933	3.212912
**S100A9**	0.505054	0.269118	0.879279	0.266268	0.876931
**ZSCAN18**	0.540076	0.280863	0.949427	0.292462	0.972441
**F2RL1**	1.912172	1.074282	3.692009	1.054937	3.623047
**EGFL6**	0.463106	0.235597	0.83433	0.23606	0.841956
**ADGRG6**	2.712558	1.593227	5.241306	1.474584	4.89997
**COL8A2**	0.508989	0.271427	0.83458	0.282756	0.855419
**PDE4D**	2.122275	1.220449	4.164362	1.133518	4.039384
**EGFR**	1.794727	1.041885	3.212464	1.008335	3.136975
**ABCA4**	0.526956	0.278465	0.94388	0.27772	0.943084
**SPRR2B**	0.570465	0.306516	0.999423	0.297087	0.996577
**ATF3**	1.861422	1.097457	3.510935	1.081672	3.393476
**CTTN**	2.273275	1.307902	4.512247	1.175267	4.237856

### Establishment of a 15-Gene Signature by Least Absolute Shrinkage and Selection Operator Regression and Stepwise Multivariate Cox Hazard Ratio Regression Modeling

**Figure 1:**
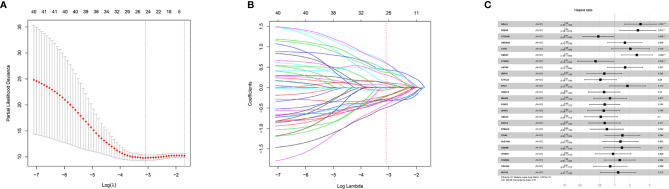
LASSO regression and 25-gene model construction. **(A)** Tuning parameter (λ) selection using LASSO penalized logistic regression with 10-fold cross-validation, **(B)** LASSO model coefficients and, **(C)** 25-Gene model obtained *via* LASSO feature selection.

**Figure 2:**
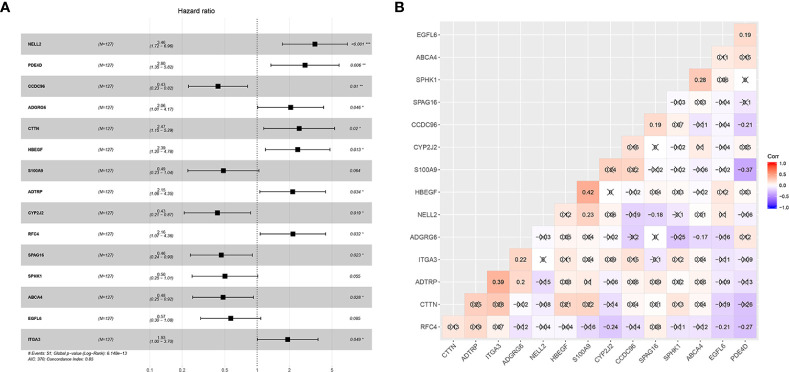
Establishment of the 15-mRNA signature and collinearity assessment. **(A)** Forest plot of the 15-mRNA prognostic model constructed by COX stepwise regression and **(B)** Co-expression analysis of the 15-gene signature.

Forty-one differentially-expressed cancer-related genes, selected by univariate Cox regression analysis, were incorporated in the LASSO regression analysis. The regularization parameter (lambda) was set to log.lambda.min. Then 25 genes were considered statistically significant and included into the LASSO regression model (Shown in **Figure 1**). These 25 genes were incorporated in the stepwise multivariate Cox hazard ratio regression to select the best model for predicting overall survival of OTSCC, and resulted in a 15-gene prognostic signature involving *ADTRP, ITGA3, RFC4, CCDC96, CYP2J2, NELL2, SPHK1, SPAG16, HBEGF, S100A9, EGFL6, ADGRG6, PDE4D, ABCA4*, and *CTTN* (**Figure 2A**). The results of the stepwise multivariate Cox hazard ratio regression are summarized in **Table 3**. Co-expression analysis, employed to assess the collinear relationship of the 15-gene prognostic signature, indicated the absence of collinearity problems (**Figure 2B**).

**Table 3:** 15-gene signature selected by stepwise multivariate Cox regression.

Gene Symbol	Description	Entrez ID	HR (95% CI)	*p*
**NELL2**	Neural Epidermal Growth Factor-Like 2	4753	3.46 (1.72–6.96)	<0.001
**PDE4D**	Phosphodiesterase 4D	5144	2.80 (1.35–5.82)	0.006
**CCDC96**	Coiled-Coil Domain-Containing Protein 96	257236	0.43 (0.23–0.82)	0.01
**ADGRG6**	Adhesion G Protein-Coupled Receptor G6	57211	2.06 (1.01–4.17)	0.046
**CTTN**	Cortactin	2017	2.47 (1.15–5.29)	0.02
**HBEGF**	Heparin Binding EGF Like Growth Factor	1839	2.39 (1.20–4.78)	0.013
**S100A9**	S100 Calcium Binding Protein A9	6280	0.49 (0.23–1.04)	0.064
**ADTRP**	Androgen Dependent TFPI Regulating Protein	84830	2.15 (1.06–4.35)	0.034
**CYP2J2**	Cytochrome P450 Family 2 Subfamily J Member 2	1573	0.43 (0.21–0.87)	0.019
**RFC4**	Replication Factor C Subunit 4	5984	2.16 (1.07–4.36)	0.032
**SPAG16**	Sperm Associated Antigen 16	79582	0.46 (0.24–0.90)	0.023
**SPHK1**	Sphingosine Kinase 1	8877	0.50 (0.25–1.01)	0.055
**ABCA4**	ATP Binding Cassette Subfamily A Member 4	24	0.48 (0.25–0.92)	0.028
**EGFL6**	EGF Like Domain Multiple 6	25975	0.57 (0.30–1.08)	0.085
**ITGA3**	Integrin Subunit Alpha 3	3675	1.93 (1.00–3.70)	0.049

**Figure 3:**
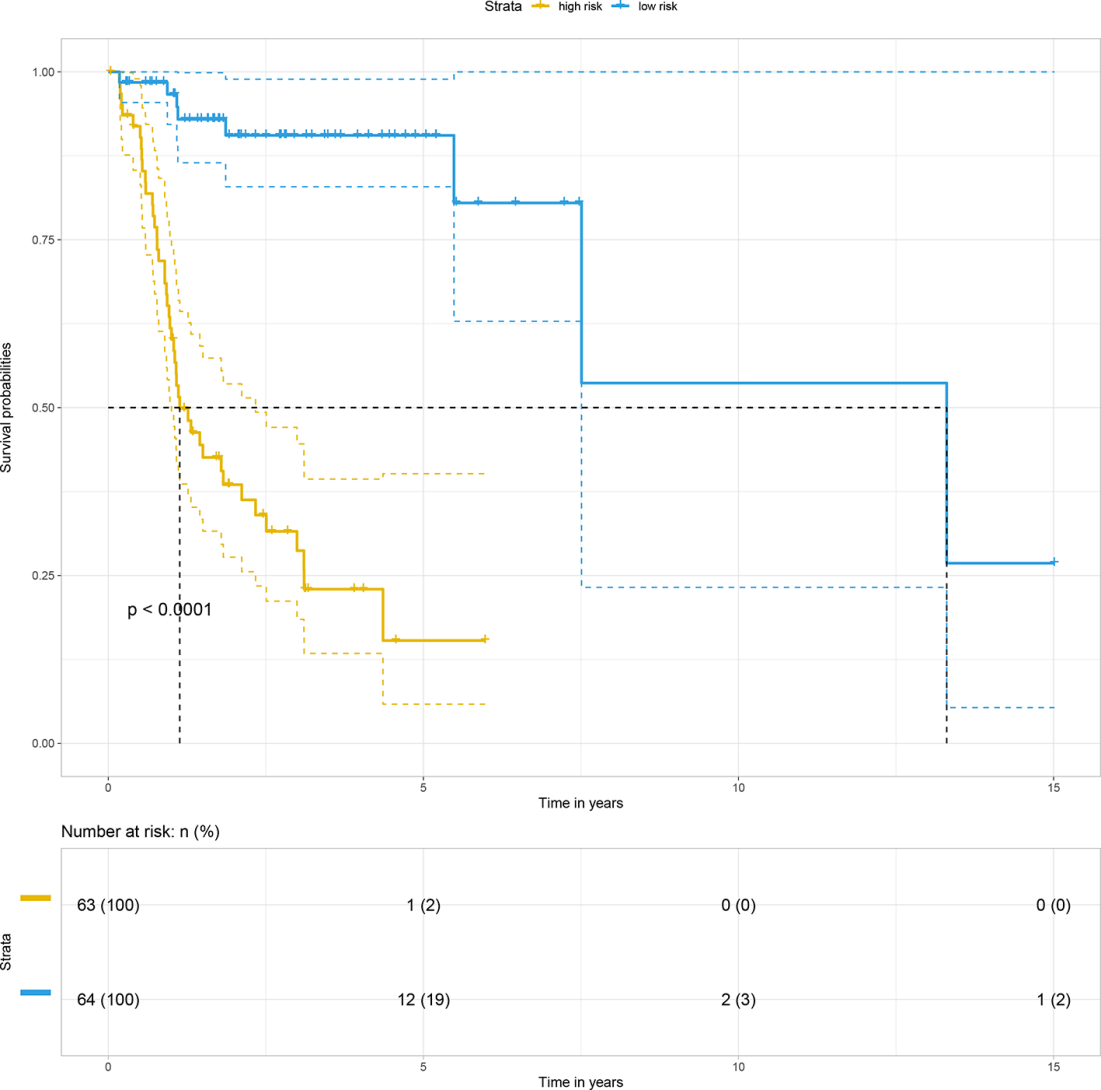
Kaplan–Meier survival curve based on risk score model for the training cohort.

Subsequently, by using the median risk score of the 15-gene signature as a cutoff value, 127 non-distant metastatic OTSCC patients were divided into a high-risk group (n = 63) and a low-risk group (n = 64). Kaplan-Meier survival analysis indicated the high-risk group had significantly poorer overall survival than the low-risk group (*p* < 0.0001) (**Figure 3**).

### Validation of the 15-Gene Signature for Prediction of Non-Distant Metastatic Oral Tongue Squamous Cell Carcinoma Overall Survival

**Figure 4:**
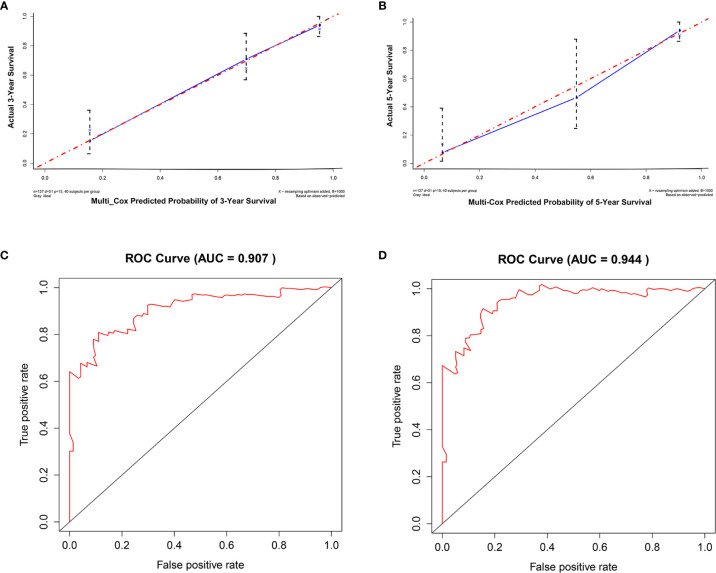
Internal validation of the 15-mRNA signature. **(A)** Calibration plot prediction for 3-year overall survival of OTSCC for the training cohort, **(B)** calibration plot prediction for 5-year overall survival of OTSCC for the training cohort, **(C)** receiver operating characteristic curve with 3-year AUC for the training cohort, and **(D)** receiver operating characteristic curve with 5-year AUC for the training cohort.

For internal validation, the accuracy and reliability of the 15-gene prognostic signature was validated by three different methods. First, the discrimination ability of the 15-gene signature prognostic model was assessed using a C-index. In the present study, the C-index was 0.849, indicating excellent prediction accuracy of the fitted model. Second, the calibration plot-predicted 3-year and 5-year overall survival of M0 OTSCC, built to internally validate the 15-gene signature, performed very well compared with the ideal model (**Figures 4A, B**). Finally, the prediction performance of the 15-gene signature for M0 OTSCC patients, evaluated using the area under receiver operating characteristic curve (ROC AUC), showed that our prognostic model provided excellent prediction performance (3-year AUC = 0.907 and 5-year AUC = 0.944) (**Figures 4C, D**).

**Figure 5:**
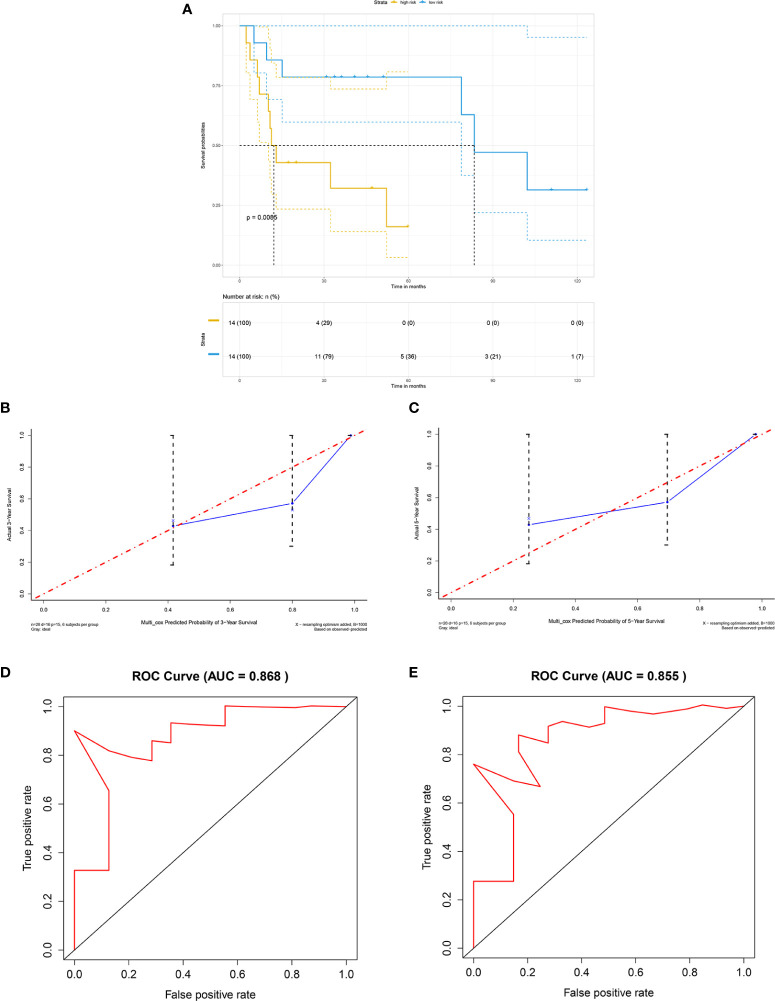
External validation by GEO cohort. **(A)** Kaplan-Meier survival curve based on risk score model for the external validation cohort GSE41116, **(B)** calibration plot prediction for 3-year overall survival of OTSCC for the external validation cohort GSE41116, **(C)** calibration plot prediction for 5-year overall survival of OTSCC for the external validation cohort GSE41116, **(D)** receiver operating characteristic curve with 3-year AUC for the external validation cohort GSE41116, and **(E)** receiver operating characteristic curve with 5-year AUC for the external validation cohort GSE41116.

For external validation, GSE41116 was employed. Consistent with the training set, survival analysis showed that in the external validating set, the high-risk group had significantly poorer overall survival than the low-risk group (*p* = 0.0086) (**Figure 5A**). Moreover, the external validating set yielded a C-index of 0.804, with a 3-year AUC of 0.868 and 5-year AUC of 0.855, which implied excellent prediction accuracy of the fitted model (**Figures 5D, E**). In addition, the calibration plot predicted 3-year and 5-year overall survival of non-distant metastatic OTSCC patients and also showed excellent performance when compared with the ideal model (**Figures 5B, C**).

### The 15-Gene Signature Serves as an Independent Predictor of Overall Survival in Non-Distant Metastatic Oral Tongue Squamous Cell Carcinoma Patients

**Figure 6:**
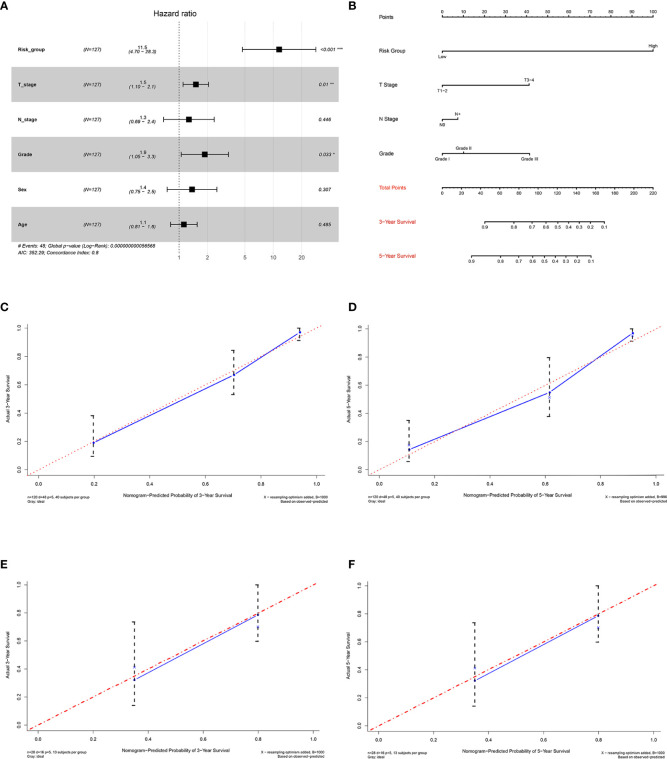
Establishment and validation of the prognostic nomogram. **(A)** Forest plot integrating the 15-gene signature and clinicopathological parameters, **(B)** nomogram for predicting 3-year and 5-year overall survival of OTSCC patients, **(C)** 3-year calibration plot for internal validation of the prognostic nomogram, **(D)** 5-year calibration plot for internal validation of the prognostic nomogram, **(E)** 3-year calibration plot for external validation of the prognostic nomogram by GSE41116, and **(F)** 5-year calibration plot for external validation of the prognostic nomogram by GSE41116.

To evaluate the independent effect of the 15-gene signature on overall survival for M0 OTSCC patients, we used a stepwise multivariate COX regression analysis to adjust clinicopathological parameters, including age, sex, T stage, N stage, and histologic grade. The results indicated that the predictive ability of the 15-gene signature was independent of other clinicopathological factors for overall survival of M0 OTSCC patients (HR = 11.5, 95% CI = 4.70–28.3, *P* < 0.001) (**Figure 6A**).

### Establishment and Internal Validation of the Prognostic Nomogram for Predicting Overall Survival in Non-Distant Metastatic Oral Tongue Squamous Cell Carcinoma Patients

To ensure the robustness and practicability of the 15-gene prognostic model, a prognostic nomogram for predicting overall survival in non-distant metastatic OTSCC patients was established (**Figure 6B**). Independent prognostic factors, such as risk group, grade, T stage, and N stage, were included in the nomogram. Clinicians could easily predict the 3-year and 5-year overall survival rate based on the sum of all scores of individual prognostic factors in the nomogram. The nomogram was internally validated by computing the bootstrap C-index and calibration plot. The predicted 3-year and 5-year overall survivals, from the bootstrapped calibration plots, were excellent compared with the ideal model (**Figures 6C, D**).

## Discussion

In the current study, we established a 15-gene prognostic signature, for predicting overall survival of non-distant metastatic OTSCC, that involved *ADTRP, ITGA3, RFC4, CCDC96, CYP2J2, NELL2, SPHK1, SPAG16, HBEGF, S100A9, EGFL6, ADGRG6, PDE4D, ABCA4*, and *CTTN*. The prediction ability of this 15-gene signature was independent of other clinicopathological factors, with an HR of 11.5 (95% CI: 4.70–28.3). Moreover, three different validation methods were utilized to assess the accuracy and reliability of the 15-gene prognostic model, including C-index, calibration plot, and ROC curve. Our signature possesses excellent prediction accuracy of the fitted model, with a C-index of 0.849, 3-year AUC of 0.907 and 5-year AUC of 0.944 for internal validation, and with a C-index of 0.804, 3-year AUC of 0.868 and 5-year AUC of 0.855 for external validation. Based on our findings, we proposed the 15-gene signature for potential survival prediction in non-distant metastatic OTSCC patients. In addition, to ensure the robustness and practicability of the 15-gene prognostic model, a prognostic nomogram integrating risk group, grade, T stage, and N stage was established. Clinicians can conveniently predict the 3-year and 5-year overall survival of M0 OTSCC patients based on the sum of all scores of the individual prognostic factors in the nomogram. Furthermore, validation by computing the bootstrap C-index and calibration curve indicated good performance by the nomogram.

In recent years, more and more multiple-biomarker signatures and prognostic nomograms have been established and utilized for clinical decision-making and prognostic evaluation for different type of cancers (5, 10–12). However, few biomarker-based signatures and prognostic nomograms have been studied in relationship to OTSCC (13–15). After studying tumor characteristics of 1,617 patients with cancer of the oral cavity, Montero et al. constructed a prognostic nomogram for oral cavity cancer patients (16). Their nomogram included several important clinicopathological variables such as age, sex, race, alcohol and tobacco use, oral cavity subsite, invasion of other structures, tumor size, and clinical nodal status, but unfortunately, did not include biomarkers. The concordance index of their model was only 0.67. In 2016, Bobdey et al. successfully developed a prognostic nomogram for prediction of the 5-year OS for oral cavity cancer patients (17). Their nomogram integrated important clinicopathological factors, including age, comorbidities, clinical lymph node status, stage of disease, tumor thickness, differentiation, and perineural invasion, and gave a C-index of 0.7263. However, similar to Montero’s study, biomarkers were not included in their prognostic model. In 2017, after integrated analysis of five GEO data sets and one TCGA data set, Qiu et al. (15). obtained a 16-mRNA prognostic signature for prognosis of OTSCC, but the C-index for predicting OS was only 0.652, indicating low predictive power. In their 16-mRNA prognostic signature, *CD69, CDS2, CPE, EVI2A, FAM69A, GUSB, HNF1B, ITM2A, MBD4, NPY, RGS5, SEL1L3, SELL, SMG1, SNX4*, and *ZC3H3* were included, which were totally different from our 15-mRNA signature. The possible reasons for this difference might be as follows. First, Qiu et al. used five GEO data sets as training set to identify differentially expressed genes. Different from Qiu et al., we used TCGA data set as training set. Second, genes with an average expression less than 100 were excluded in our study. Third, we conducted an oncogenic signature enrichment analysis before our prognostic model was established to ensure only cancer-related genes would be selected into our model.

When compared to the previous studies, there are several advantages in our study. First, we conducted an oncogenic signature enrichment analysis before our prognostic model was established to ensure only differentially-expressed cancer-related genes would be selected into our model. Thus, we avoided accidental inclusion of differentially-expressed non-cancer-related genes in order to make our prognostic model more reliable. Second, small sample size could magnify the error of analysis. In the current study, a bootstrap resampling technique was employed during univariate analysis to expand the sample size and make our results more convincing. Third, LASSO regression analysis was utilized in our study before stepwise multivariate Cox regression was used, which was helpful for minimizing genetic collinearity problems. Fourth, our prognostic model yielded a high C-index (C-index = 0.849) and AUC (3-year AUC of 0.907 and 5-year AUC of 0.944), indicating better prognostic prediction than previous models. Fifth, an independent GEO data set was used for external validation, which made our fitted model more reliable. Last but not least, a prognostic nomogram integrating a multi-mRNA signature and clinicopathological variables was established to facilitate the clinical use of our 15-mRNA signature.

Several limitations should be considered while reviewing our results. First, there are only 13 normal tongue samples available in TCGA, and the small sample size of normal tongue tissue was underpowered to yield a credible result of differentially-expressed gene analysis. Second, only non-distant metastatic cases were included in our study. Hence, the results of this study may not be applicable to the OTSCC patients with distant metastasis. Third, the fitted model was constructed based on existing data from public database. Unfortunately, some clinicopathological risk factors, such as depth of invasion, perineural invasion, vascular invasion, and so on, are unavailable from the existing data. Fourth, three different methods had been utilized for internal validation and external validation of our 15-gene prognostic signature, but the external validating cohort in the present study contained only 28 samples. An external validating cohort with larger sample sizes will be more helpful to confirm our findings. Finally, the current study is a retrospective study. A prospective randomized clinical trial is needed before the fitted model could be used in clinical practice.

## Conclusions

In conclusion, our results demonstrate our 15-gene signature was independently associated with overall survival in non-distant metastatic OTSCC. Moreover, the prognostic nomogram integrating the 15-gene signature and clinicopathological factors has potential to be developed as a prognostic tool.

## Data Availability Statement

The original contributions presented in the study are included in the article/**Supplementary Material**. Further inquiries can be directed to the corresponding author.

## Author Contributions 

All authors contributed to the design of the study. ML, LT, BL, XS, LX, HP, and DH contributed in the acquisition of the data, manuscript preparation, and revision. All authors participated in study planning, data interpretation, and writing the manuscript. All authors contributed to the article and approved the submitted version.

## Funding

This project was supported by the National Natural Science Foundation of China (NSFC) (Grant No. 31770876), the Guangdong Medical Research Foundation, China (Grant No. A2019424), and the Shantou Science and Technology Project, Guangdong Province, China (Grant No. 180723154011142).

## Conflict of Interest

The authors declare that the research was conducted in the absence of any commercial or financial relationships that could be construed as a potential conflict of interest.
