# Supplementary figures and images for: A 15-Gene Signature and Prognostic Nomogram for Predicting Overall Survival in Non-Distant Metastatic Oral Tongue Squamous Cell Carcinoma
Source: Front Oncol. 2021 Mar 9;11:587548. doi: 10.3389/fonc.2021.587548 (PMC7985252; doi:10.3389/fonc.2021.587548)

# Volcano

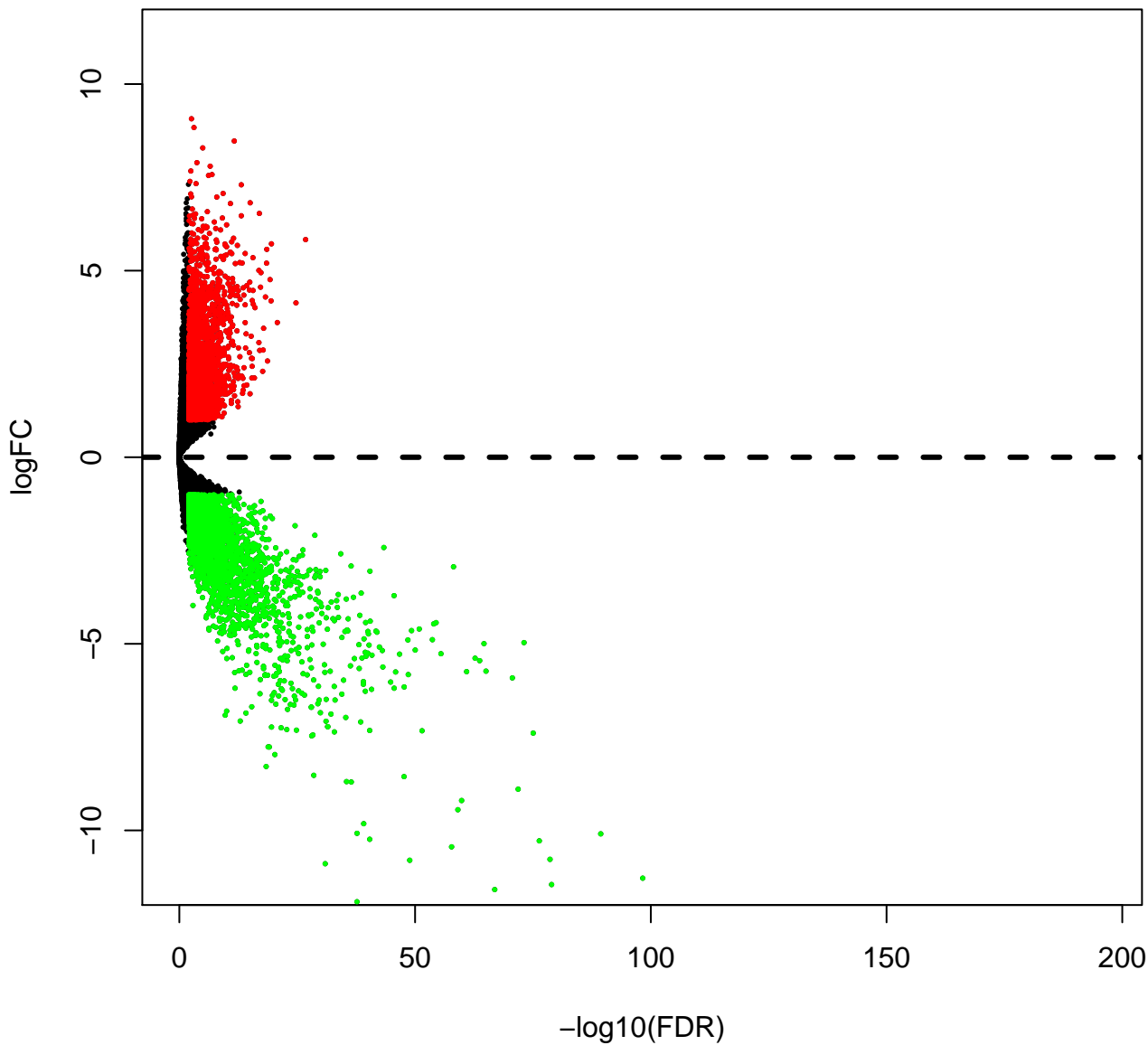

Supplement: Supplementary file 2 [file DataSheet_2.pdf]
