# Supplementary material for: A 15-Gene Signature and Prognostic Nomogram for Predicting Overall Survival in Non-Distant Metastatic Oral Tongue Squamous Cell Carcinoma
Source: Front Oncol. 2021 Mar 9;11:587548. doi: 10.3389/fonc.2021.587548 (PMC7985252; doi:10.3389/fonc.2021.587548)
Supplement: Supplementary file 1 [file DataSheet_1.pdf]

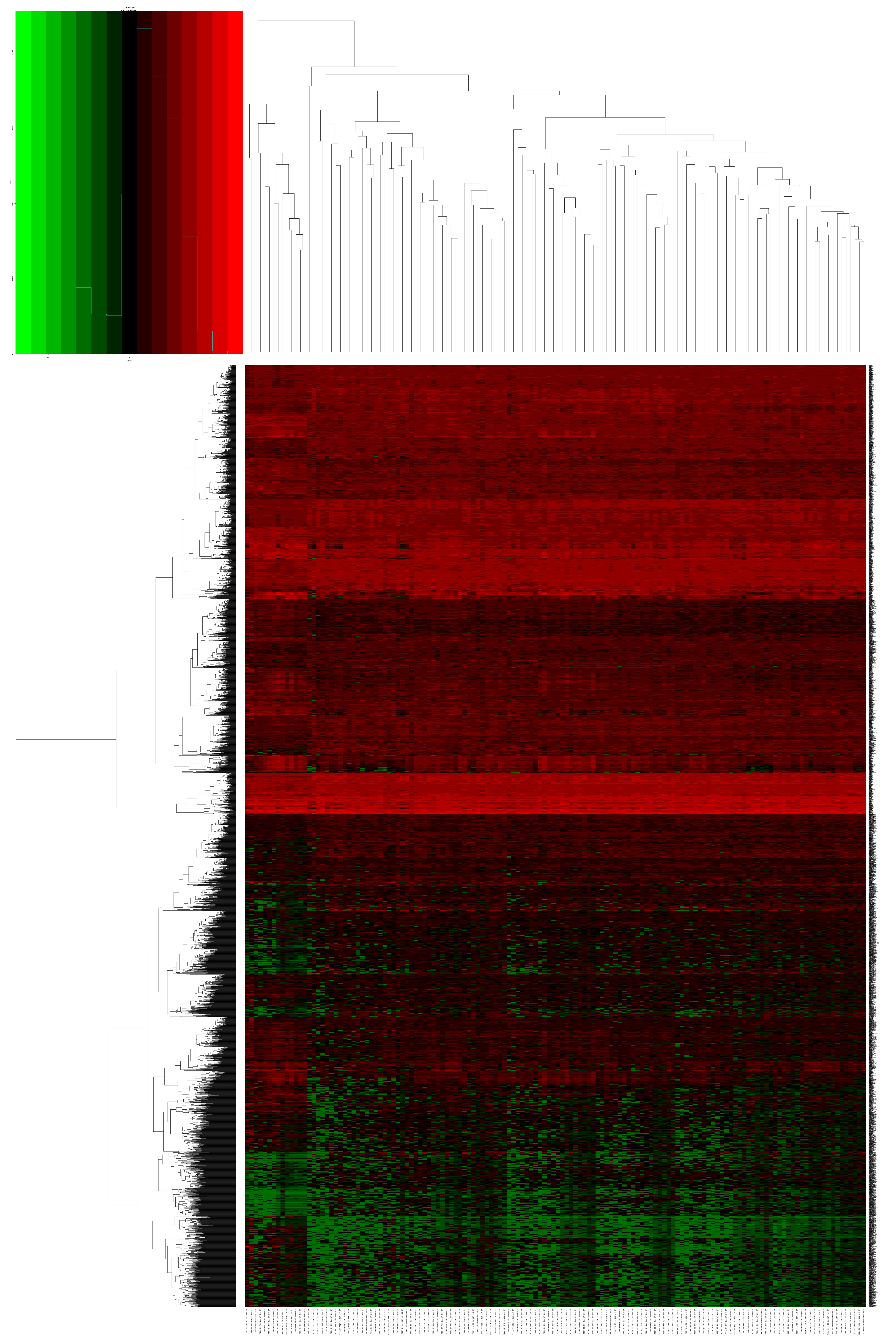
Color Key
and Histogram
Count
2e+05
150000
1e+05
50000
0
−5
0
5
Value
